# Current Data on Risk Factor Estimates Does Not Explain the Difference in Rates of Melanoma between Hispanics and Non-Hispanic Whites

**DOI:** 10.1155/2016/2105250

**Published:** 2016-03-22

**Authors:** Sonia Kamath, Kimberly A. Miller, Myles G. Cockburn

**Affiliations:** ^1^Department of Dermatology, Keck School of Medicine of the University of Southern California (USC), 1200 N State Street, Room 3250, Los Angeles, CA 90033, USA; ^2^Department of Preventive Medicine, Keck School of Medicine of USC, 2001 N. Soto Street, Suite 318-A, Los Angeles, CA 90032, USA

## Abstract

United States Hispanics have seven times lower melanoma incidence rates than non-Hispanic whites (NHW). It is unclear whether this difference can be explained solely by phenotypic risk factors, like darker skin, or whether modifiable risk factors, like sun exposure, also play a role. The purpose of this paper is to summarize what is currently known about melanoma risk factors among Hispanics and NHWs, and whether or not those differences could explain the difference in melanoma incidence. Through literature review, relative risks and prevalence of melanoma risk factors in Hispanics and NHWs were identified and used to calculate the expected rate in Hispanics and rate ratio compared to NHWs. We found that melanoma risk factors either have similar frequency in Hispanics and NHWs (e.g., many large nevi) or are less frequent in Hispanics but do not explain a high proportion of disease variation (e.g., red hair). Considering current knowledge of risk factor prevalence, we found that melanoma incidence rates in the two groups should actually be similar. Sun exposure behavior among Hispanics may contribute to the explanation for the 7-fold difference in melanoma rates. Currently, limited data exist on sun exposure behavior among Hispanics, but possibilities for improving primary prevention by further studying these practices are substantial.

## 1. Introduction

Hispanics are the largest ethnic group in the United States, and over the last few decades, rates of melanoma among Hispanics have steadily risen [1, 2]. In California, where Hispanics comprise almost 40% of the population, increases in melanoma have occurred for tumors with the worst prognosis [3–5]. Hispanics are diagnosed with melanoma at later stages than non-Hispanic whites (NHW), leading to increased likelihood of metastasis and higher mortality [3, 6, 7]. Still, the rates of melanoma in the Hispanic population remain approximately seven times lower than in the NHW population in California [3]. This difference in melanoma incidence between Hispanics and NHWs has traditionally been attributed to the protective effects of darker skin pigmentation [5]. However, Hispanics have significant heterogeneity of skin color and phototype (including light skin color), and it is well known that ultraviolet (UV) exposure plays a role in the development of melanoma regardless of skin type [8, 9]. In fact, it is unclear whether key melanoma risk factors, including increased numbers of nevi, freckling, poor tanning ability, and fair complexion, differ substantially between Hispanics and NHWs [10, 11]. Few studies describe melanoma risk factors in Hispanics, or those risks relative to NHWs [12]. Most studies have focused on determining relative risks (RRs) for melanoma in NHWs, and few estimate melanoma burden attributable to each risk factor [13, 14].

In this review, we examined what is currently known about the risk factors for melanoma and their distribution among Hispanics and then calculated expected rate ratios (NHW compared to Hispanic) for each risk factor based on published estimates of relative risk and risk factor prevalence in the two groups. We found that, based on what is currently known about the risk factors, the rates of melanoma among Hispanics and NHWs should be quite similar, and the fact that they are not similar may present an opportunity to investigate modifiable risk factors for melanoma—for which very little data exists in the Hispanic population—and to improve prevention efforts.

## 2. Materials and Methods

### 2.1. Defining the Risk Factors for Melanoma

For summary relative risk (RR) measures for melanoma risk factors, we used a comprehensive meta-analysis by Gandini et al. of 83 observational studies through September 2002 [20–22]. A PubMed search covering observational studies through April 2013 was conducted to provide a RR range for those risk factors not included in the meta-analysis.

Phenotypic risk factors for melanoma in the meta-analysis include blonde or red hair, many freckles, nevus counts, and Fitzpatrick phototype I or II. For freckles, the RR presented in the meta-analysis compared estimates for high versus low density of freckles. We focus on risk associated with increased numbers of common nevi, since dysplastic nevi have been noted to occur specifically in melanoma-prone families [22, 23]. Sunburn and sun exposure represent important risk factors for melanoma. Sun exposure was divided into total lifetime, intermittent, and chronic exposure. Intermittent sun exposure included estimates based on recreational activities, while chronic sun exposure was assessed as occupational exposure. Where multiple levels of exposure were reported, the highest level was included [20, 21].

For counts of large nevi, history of one or more childhood sunburns, and history of one or more lifetime sunburns, RRs were not summarized in the meta-analysis, so a literature search was conducted. Combinations of the following keywords and MeSH terms were used in PubMed: melanoma, etiology, epidemiology, prevention and control, risk factors, case-control studies, cohort studies, cross-sectional studies, nevus, skin pigmentation, skin color, hair color, sun exposure, sunlight, ultraviolet rays, sunburn, suntan, and sunbathing. Included studies were limited to those in English and those examining adult populations. Duplicates, reviews, and irrelevant articles were excluded. Other studies were excluded for ineligible study design (e.g., case series) (14 studies); using outcome of second primary melanoma (11 studies); not being independent of other included studies (3 studies); presenting data by gender, body site, or melanoma subtype (16 studies); or focusing on a restricted age group (1 study). An exception was made for Qureshi et al. and Han et al., as although they are based on the same cohort, the risk factors examined in each are different [24, 25].

We extracted study location, study design, number and source of cases and controls, age of study population, definitions and categories of risk factors, prevalence estimates, RR or odds ratio (OR) estimates, 95% confidence interval (CI), and variables for which statistical adjustment was done. For articles that presented multiple estimates, we recorded the one adjusted for the most confounders. For risk factors not included in the Gandini meta-analysis, the RR range represents estimates from all included studies, and the median of this range is reported in place of a summary statistic. For other risk factors, the RR range is provided as an update of studies published after 2002.

### 2.2. Calculating the Expected Rate Ratio of Melanoma in Non-Hispanic Whites Compared to Hispanics

In order to compare the melanoma rates between Hispanics and NHWs in California, we obtained California population-based risk factor prevalence data wherever possible. The California Health Interview Survey (CHIS) provides information on sunburn prevalence in NHWs and Hispanics [18]. We obtained the prevalence of having more than three lifetime episodes of sunburn, as this was the highest exposure level reported in the majority of papers. The Los Angeles Multiethnic Cohort provided the prevalence of sunburn and red or blonde hair color in Hispanics and NHWs [15]. Prevalence of additional phenotypic characteristics and large nevi was obtained from the California Twin Program [16]. For phototype and chronic sun exposure, estimates were used from the National Health and Nutritional Examination Surveys (NHANES) and the National Health Interview Survey (NHIS) [17, 19]. Lastly, because of the lack of population-based prevalence information on sun exposure and counts of common nevi, we calculated a weighted average of the prevalence reported among controls of the included population-based studies.

We used the reported RR for each melanoma risk factor and the risk factor prevalence among Hispanics, to calculate the expected rate of melanoma incidence among Hispanics, and the ratio compared to that in NHWs. We present the rate ratio using an incidence rate of 29 per 100,000 in NHWs (observed in males in California), for which the comparable observed rate ratio is 7.25 (the comparable melanoma incidence rate in Hispanic males in California is approximately 4 per 100,000) [3].

Because so little data are available on the RR of melanoma risk factors specific to Hispanic populations, we have assumed that the RR in Hispanics is the same as in NHWs (discussed below). Where prevalence of risk factors was unknown in Hispanic populations, we could not calculate the expected rate ratio.

## 3. Results and Discussion

### 3.1. Does the Prevalence of Melanoma Risk Factor Phenotypes Explain the Lower Rate of Melanoma in Hispanics?

Table 1 summarizes the risk factors, RR ranges, prevalence ranges, and calculated rate ratios [15–19, 24–95]. Of the risk factors for which an expected rate ratio could be calculated, skin phototype I or II was responsible for the highest expected rate ratio, but this was only 1.40. Blonde hair was associated with the second highest expected rate ratio (1.26) in NHWs compared to Hispanics. Having many large nevi, although carrying RR of 3.05, demonstrated an expected rate ratio of 1.04 (because the prevalence of large nevi is similar in NHW and Hispanics), and high chronic sun exposure accounted for no difference in expected melanoma rates between Hispanics and NHWs. In both cases the expected rate ratio was near null because of the similarity of prevalence of the risk factor in Hispanics and NHWs.

Most studies comparing rates of melanoma in Hispanics and NHWs focus on delayed diagnosis and overall worse outcomes among Hispanics, while few specifically compare risk factors. Our review confirms that Hispanics do have lower prevalence of some of the major melanoma risk phenotypes, blonde or red hair color, Fitzpatrick skin type I or II, strong history of sunburn, and many large nevi. However, these risk factors are not sufficiently rare in Hispanics to explain their lower rate of melanoma compared to NHWs, largely because there is overlap in the prevalence of the risk factors between Hispanics and NHWs.

### 3.2. Could Sun Exposure Behavior Explain the Lower Rate of Melanoma in Hispanics?

Hispanics may have a reduced melanoma risk if they practice better sun protection. Recent studies highlight differences in method of sun protection among subpopulations of Hispanics. Specifically, English-acculturated Hispanics display more sunscreen use but less use of sun protective clothing, while Spanish-acculturated Hispanics are more likely to wear sun protective clothing without sunscreen [19, 96]. Additionally, English-acculturated Hispanics are more likely to have had a sunburn in the past year compared to those less acculturated, consistent with data showing that sunscreen may not be as effective as sun protective clothing at preventing sunburn [19, 97]. Given the diversity of sun protective practices among Hispanics, studying the relationship between sun exposure behavior and melanoma stratified by level of acculturation would help clarify whether safer sun exposure behavior accounts for overall low rates of melanoma in this population.

Data from the Behavioral Risk Factor Surveillance System (BRFSS) shows that overall a smaller percentage of Hispanics reported having at least one sunburn in the preceding year compared to NHWs. Hispanics who reported having at least one sunburn in the past year were more likely to have had* only* one sunburn, compared to NHWs [98]. However, BRFSS data also show that sunburn among Hispanics is relatively common, which could contribute to their increasing incidence of melanoma. Compared to NHWs, Hispanics do have higher prevalence of chronic sun exposure, shown to be protective against melanoma compared to intermittent exposure [9, 10, 99].

### 3.3. Could Genetic Effects Explain the Lower Rate of Melanoma in Hispanics?

A multitude of genetic factors, if substantially different in the two populations, could either protect Hispanics or make NHWs more susceptible to melanoma. Six loci have been implicated in melanoma susceptibility. While five of them represent genes involved in melanin production (*MC1R*,* TYR*,* TYRP1*,* SLC45A2*, and* ASIP*), the sixth locus is thought to represent two genes, both involved in nevus formation (*CDKN2A* and* MTAP*) [10, 100]. The* CDKN2A* gene is inherited in an autosomal dominant fashion and is associated with familial melanoma, which represents only 5 to 12% of melanoma cases [101]. The prevalence of these genotypes in Hispanics is unknown.

The* MC1R* gene codes for the melanocortin-1 receptor. Weak signaling of this receptor due to inactivating polymorphisms in the* MC1R* gene results in production of the red pigment pheomelanin (“red hair/fair skin” phenotype), instead of the brown/black eumelanin [102]. It has recently been shown that pheomelanin may be carcinogenic itself, independent of UV radiation, particularly in the context of an activating mutation in the kinase BRAF (the most common driver mutation in melanoma). Specifically, mice with an inactivating* MC1R* mutation and the BRAF mutation had red fur and developed melanoma, while mice who did not produce pheomelanin did not develop melanoma, despite having the BRAF mutation [103]. However, given the overlap in skin types between Hispanics and NHWs and the near-null expected rate ratio for skin type in our analysis, variations in the population prevalence of* MC1R* inactivating polymorphisms and pheomelanin are unlikely to fully explain the large difference in melanoma incidence.

*BRAF* mutations are more common in melanomas occurring in skin intermittently exposed to high amounts of sun compared with melanomas arising on unexposed or chronically exposed skin [104]. It is possible that fewer Hispanics have* BRAF* mutations, which could help to explain their lower incidence of melanoma. Since most clinical trials have been conducted in NHWs, there is a paucity of literature on genetic mutations in other populations [105]. Further studies are necessary to elucidate the role of genotype in the risk for melanoma among Hispanics.

### 3.4. Limitations

The observation that Hispanics have more advanced melanomas at diagnosis than NHWs could be explained by lower awareness of melanoma in this population, resulting in lower likelihood of seeking care [106–109]. This presents the possibility that true rates of melanoma in the Hispanic population are higher than observed and that there is poorer detection of cases. However, 35.4 percent of tumors in Hispanic males in California are “thick” (>1.5 mm) at diagnosis, compared with 24.4 percent of tumors in NHWs, so the lack of detection of thin tumors through screening in Hispanics alone is unlikely to explain a 7-fold difference in the overall incidence rate [2].

We make a number of assumptions in our calculations, which are done for illustrative purposes. First, we assume that the RR for each risk factor is the same in Hispanics and NHWs. This is because there is little data assessing melanoma risk factors specifically in Hispanics. It is possible that RRs for melanoma risk factors differ for NHWs and Hispanics but that remains to be shown. For illustrative purposes, we have used incidence data only from NHW and Hispanic males in California, but the rate ratios for comparison among females in California provide similar results. Finally, we considered all invasive melanomas together, though the distributions of the various melanoma histologic subtypes are known to differ between NHW and Hispanics [5, 9]. However, risk factors for nodular and superficial spreading melanoma do not vary among NHWs, and there is limited data on other histology-specific risk factors [110].

## 4. Conclusions

To determine effective methods of primary prevention, it is useful to investigate characteristics associated with lower risk of melanoma. To that end, the melanoma experience of Hispanics, who have at least 7-fold lower risk of melanoma than NHWs, might provide clues to improved melanoma prevention. This review highlights the limited data on melanoma risk phenotypes in Hispanic populations; what is currently known about the differences in their prevalence between Hispanics and NHWs inadequately explains a 7-fold difference in melanoma rates.

While genotype may vary between Hispanics and NHWs, there is insufficient data about melanoma risk genes in Hispanic populations to attribute their lower melanoma rates to genetic factors alone. The association between genotype and melanoma risk phenotype might lead us to expect greater differences in skin type and nevi prevalence between Hispanics and NHWs if differentially expressed genetic factors explained the 7-fold difference in melanoma incidence.

While little data exist comparing sun exposure behaviors in Hispanics and NHWs, current data show that Hispanics sunburn less frequently. If sun exposure behaviors help protect Hispanics from melanoma, there could be great potential for improving prevention in all populations through behavioral change. Once the sun exposure behaviors of Hispanics are more clearly understood, prevention messages might be improved. The lower rate of melanoma in Hispanics should be formally investigated in future studies, providing direct comparison of measured risk factors in Hispanics versus NHWs and accounting for any differences in sun exposure behaviors, phenotypic and genotypic characteristics. Such an approach may better inform our methods of melanoma prevention in both Hispanics and NHWs.

## Figures and Tables

**Table 1:** Summary of relative risks (RR) and prevalence of risk factors.

Risk factor	Category	RR^a^	95% CI	RR range	Prevalence (%)		Expected rate ratio^b^	Source
					NHW	Hispanic	(Observed = 7.25)	
Hair color	Blonde	1.96	1.41–2.72	0.45–4.13	13.60–46.80	0.90–3.60	1.26	Park et al., 2012 [15] Cockburn et al., 2007 [16], CTP^c^
	Red	3.64	2.56–5.37	1.73–4.94	3.10–3.20	0.30–1.30	1.06	Park et al., 2012 [15] Cockburn et al., 2007 [16], CTP

Phototype	Fitzpatrick type I	2.09	1.67–2.58	2.36–2.64	5.00–7.60	1.00–3.30	1.04	Lin et al., 2012 [17] Galindo et al., 2007 [8]
	Fitzpatrick type II	1.84	1.43–2.36	1.82–4.13	26.70–39.00	10.70–12.00	1.16	Lin et al., 2012 [17] Galindo et al., 2007 [8]
	Fitzpatrick type I or II	2.99	1.75–5.12	1.31–2.90	34.30–44.00	13.00–14.00	1.40	Lin et al., 2012 [17] Galindo et al., 2007 [8]

Freckles	Many freckles	2.10	1.80–2.45	1.55–3.72	7.43 (controls)	Not available	~	

Nevi	Many nevi	4.82	3.05–7.62	1.50–6.50	11.66 (controls)	Not available	~	
	Many large nevi	3.05		1.19–5.70	5.70	3.60	1.04	CTP

Sunburn	Many sunburns (lifetime)	2.03	1.73–2.37	0.59–8.48	7.50–20.70	3.80–4.00	1.10	Park et al., 2012 [15] CHIS 2009 [18]
	Many sunburns (childhood)	2.24	1.73–2.89	1.00–6.22	13.70 (controls)	Not available	~	
	Ever had sunburn (lifetime)	1.21		1.10–5.70	61.8	21	1.08	Park et al., 2012 [15]
	Ever had sunburn (childhood)	1.47		0.90–3.56	28.19 (controls)	Not available	~	

Sun exposure	High total lifetime sun exposure	1.34	1.02–1.77	0.80–4.34	27.11 (controls)	Not available	~	
	High intermittent sun exposure	1.61	1.31–1.99	0.65–5.00	40.54 (controls)	Not available	~	
	High chronic sun exposure	0.95	0.87–1.04	0.33–2.57	5.44 (controls)	12.00	1.00	Coups et al., 2012 [19]

^a^RR represents summary statistic from meta-analysis by Gandini et al., 2005 [20–22] (I, II, and III) or median of RR range where summary statistic is not reported.

^b^Expected ratio of incidence rates (NHW/Hispanic) based on prevalence of risk factor in each population and RR provided. Median prevalence value is used when a range is provided.

^c^CTP: California Twin Program; prevalence data for NHWs is described by Cockburn et al., 2007 [16], and prevalence data for Hispanics comes from the same data set.

~Expected rate ratio cannot be calculated because prevalence in Hispanics is unknown.
